# The retinoic acid receptor alpha (*RARA*) gene is not associated with myopia, hypermetropia, and ocular biometric measures

**Published:** 2009-07-17

**Authors:** S. Veerappan, M. Schäche, K.K. Pertile, F.M.A. Islam, C.Y. Chen, P. Mitchell, M. Dirani, P.N. Baird

**Affiliations:** 1 Centre for Eye Research Australia, University of Melbourne, Royal Victorian Eye and Ear Hospital, Melbourne, Australia; 2 Vision Cooperative Research Centre, Sydney, Australia; 3 Centre for Vision Research, University of Sydney, Australia

## Abstract

**Purpose:**

The Retinoic Acid Receptor Alpha (*RARA*) gene is a potential candidate gene for myopia due to its differential expression in animal models during experimentally induced myopia. To test for whether *RARA* is associated with myopia we have undertaken a case-control study assessing for associations between *RARA* and myopia, hypermetropia, and ocular biometric measures.

**Methods:**

A total of 802 Anglo-Celtic individuals were genotyped. Five tag single nucleotide polymorphisms (tSNPs) in *RARA* with an r^2^ of 0.8 and a minor allele frequency greater than 5% were selected for genotyping. Genotype frequencies of these 5 tSNPs were compared between individuals with emmetropia and those with myopia or hypermetropia. A quantitative analysis was also performed to assess associations with ocular biometric measures including axial length, corneal curvature and anterior chamber depth.

**Results:**

We did not identify any significant association between tSNPs in *RARA* with either myopia or hypermetropia as qualitative traits. Neither did we identify any significant associations of these tSNPs with the quantitative traits of axial length, corneal curvature and anterior chamber depth.

**Conclusions:**

This is the first study to assess for associations between *RARA* and myopia, hypermetropia, and ocular biometric measures. Our findings suggest that variations in the nucleotide sequence of *RARA* are not associated with myopia, hypermetropia, or ocular biometric measures in our population.

## Introduction

Refractive errors, including myopia and hypermetropia, represent a diverse but common spectrum of eye disease associated with significant morbidity across the world [1,2]. Refractive errors occur when light rays from an object focus in front of (myopia) or behind (hypermetropia) the retina, leading to an unfocussed image. They present a considerable public health burden with a prevalence of 20-25% for myopia in Western nations and much higher rates in some South-East Asian countries up to 75%, with hypermetropia also being frequent, particularly in older subsamples [3-6]. The four major refractive components of the eye are represented by the power of the cornea, determined in part by its curvature, the depth of the anterior chamber, the power of the lens and the length of the eye (axial length) [7]. Refractive errors arise through a failure of one or more of these refractive components typically resulting in a mismatch of axial length with refractive power [8].

Myopia and hypermetropia are complex disease traits. Environmental risk factors, such as education and near-work, are known to play a role in the development of myopia but the role that these play in hypermetropia is not clear [9-12]. Nonetheless, such risk factors only explain around 12% of the observed phenotypic variance [13]. A substantial genetic role in the development of myopia is evident from familial studies indicating that children with one or both parents presenting with myopia have a 3 to 7 fold risk of developing myopia compared to children with neither parent having myopia [14,15].

Family and twin heritability studies have indicated that refractive error, as well as axial length, corneal curvature, and anterior chamber depth are all highly heritable (heritability estimates ranging from 50% to 90%) [16-20]. Moreover, genetic linkage analyses have already identified 19 chromosomal regions that might harbor myopia genes, but so far no confirmed genes have been identified from these regions [21-31]. Several studies have sought to identify causal variants in candidate genes from these regions based on a postulated biological role in myopia [32-37]. The role of genes in the development of hypermetropia is less researched despite heritability studies predicting that hypermetropia is also highly heritable [38,39].

The underlying genes causing refractive errors such as myopia has not been fully elucidated but we hypothesized that the Retinoic Acid Receptor Alpha (*RARA*) gene represents a plausible candidate. This gene has been shown to be differentially expressed in both guinea pigs and chicks during experimentally induced form-deprivation myopia [40,41]. In addition, inhibition of the synthesis of retinoic acid, the major ligand for this receptor, has been shown to reduce form-deprivation myopia [42]. RARA represents one of six receptors for retinoic acid but unlike the others it has been shown to be strongly expressed in the retina [43,44]. Given that changes in retinal gene expression are the likely origin of signals that initiate eye growth it is not unreasonable to hypothesize that *RARA* may play a role in the development of myopia [45]. In support of this, double knockout mice lacking both copies of *RARA* have a reduced eye weight and a reduced retinal area [46].

In order to further explore the possible role of *RARA* in the development of refractive errors such as myopia we have undertaken a case-control genetic association study. We have utilized a tag single nucleotide polymorphism (tSNP) approach to analyze common polymorphisms within the coding region of *RARA* and its promoter and assessed for genetic associations to myopia, hypermetropia and ocular biometry measures.

## Methods

### Subjects

Individuals with Anglo-Celtic ancestry were included in this study with ethnicity being based on the place of birth of the participant as well as their parents and grandparents, if known. Individuals with a history of other eye diseases, such as keratoconus, glaucoma, or age related macular degeneration (AMD) that could affect refraction measurements were excluded from the study. Individuals with a history of genetic disorders known to predispose to myopia, such as Stickler or Marfan syndromes, were also excluded. Individuals with greater than a 2 D difference between eyes were excluded as well as individuals where the refractive measurement of each eye fell into 2 different refraction groups. Using the above criteria we selected all relevant participants from the Genes in Myopia Study (GEM; n=570) [47], the Blue Mountains Eye Study (BMES; n=131) [48] and the Melbourne Visual Impairment Project (VIP; n=101) [49]. Only unrelated individuals were chosen. These individuals from the GEM, BMES, and VIP studies were then pooled for use in the current study. This pooling approach was necessary in order to obtain a sufficient number of cases and controls for a statistically viable genetic association study. The similarities in the methodology for obtaining ophthalmic measurements for each of these studies facilitated this pooling approach. All participants were divided into three groups, based on their refractive measurements; myopia (≤-0.50 D), emmetropia (-0.50 to +0.75 D) and hypermetropia (>+0.75 D).

Refractive measurements of the eye were obtained using an auto-refractor (Topcon RM-8800 autorefractor; Topcon, Paramus, NJ). If objective measurements were not obtained, then subjective refractive measurements using a modified version of the Early Treatment of Diabetic Retinopathy Study (ETDRS) Protocol were used. The ocular biometry measurements of axial length, anterior chamber depth and corneal curvature (average of K1 and K2) were obtained using partial coherence interferometry (IOL master; Carl Zeiss, Oberkochen, Germany). Whole blood was collected from all subjects and DNA extracted using a standard phenol-chloroform technique [50]. Ethical approval for this study was obtained from the Royal Victorian Eye and Ear Hospital (RVEEH) Human Research Ethics Committee, Melbourne, Australia, and adhered to the tenets of the Declaration of Helsinki. Before any testing, all participants provided informed consent to participate in the study.

### SNP selection and genotyping

Known SNPs within the coding region of *RARA* as well as the region encompassing 2 kb upstream of the start of exon 1 and 1 kb downstream of the stop codon were identified from the Phase II HapMap data (Release 21a). The HapMap CEU population was chosen as being the most representative ethnic population for this study. The prevalence data was then inputted into the HaploView program (version 3.32) [51] and the inbuilt *Tagger* program was used to select tSNPs. A pair-wise tagging approach, with a criteria of r^2^ >0.8 and a minor allele frequency (MAF) >5% was used, leading to five tSNPs being chosen.

All 5 tSNPs were genotyped at the Australian Genome Research Facility (Brisbane, Australia [AGRF]) using a Sequenom® Autoflex MassSpectrometer (Sequenom, San Diego, CA) according to manufacturer instructions.

### Statistical analysis

Power calculations were performed using the Quanto 1.1 software and indicated that we were able to detect a minimum Odds Ratio (OR) of 2.5 with a power of 80% assuming an equal sample size of cases and control (each of 117 high myopia and emmetropia) under an additive model with a minor allele frequency of at least 0.05. We were also able to detect an OR of 2.5 with power 80% with a minimum of 96 emmetropia and 288 hypermetropia under an additive model with a minor allele frequency of at least 0.05. Genotype frequencies were compared for each of the myopia and hypermetropia groups relative to the emmetropia group. Deviations from Hardy Weinberg Equilibrium (HWE) were assessed using a χ^2^ goodness-of-fit test. Differences in genotype frequencies, with myopia or hypermetropia as a binary trait was analyzed using an additive model by applying the linear test of trend using SPSS (version 14.0; SPSS Inc, Chicago, IL). Quantitative analysis, with axial length (AL), corneal curvature (CC), and anterior chamber depth (ACD) were undertaken using an independent samples t-test, also through SPSS (version 14.0; SPSS Inc). To minimize type 1 errors due to multiple testing, a Bonferroni correction was applied. This meant that the threshold p-value for statistical significance was 0.05/5 =0.01 for this study. Haplotype analysis was performed for each phenotype using UNPHASED [52].

## Results

### Baseline demographics

A total of 802 individuals with mean (SD) age 55.4 (13.1) and 36.3% (n=291) male were initially genotyped in this study. There were 380 subjects in the ‘myopia’ group, 116 in the ‘emmetropia’ group and 306 in the ‘hypermetropia’ group. We observed a high correlation between right and left eyes for refraction (r^2^=0.98), axial length (r^2^=0.84), corneal curvature (r^2^=1.00) and anterior chamber depth (r^2^=0.70). These correlations were statistically significant (p<0.001) and therefore only data for the right eye was used for analysis. The mean (SD) and range values for the refraction and ocular biometric measures for the right eye are given in Table 1.

**Table 1:** Baseline Ocular biometry measures for participants in the ‘high myopia’, ‘low/moderate myopia’, ‘emmetropia’, and ‘hypermetropia’ groups.

	**Spherical equivalent (D)**	**Axial length (mm)**	**Corneal curvature (D)**	**Anterior chamber depth (mm)**
**High Myopia** **(n = 117)** (< -6.00 D)	-8.57(2.39)	26.74(1.31)	44.07(1.61)	3.59(0.46)
**Low/Moderate myopia (n = 263)** (-5.99 DS to -0.50 D)	-2.61(1.46)	24.46(1.01)	44.15(1.42)	3.53 (0.40)
**Emmetropia** **(n = 116)** (-0.499 DS to +0.75 D)	0.06(0.20)	23.26(0.62)	44.16(1.33)	3.37(0.38)
**Hypermetropia** **(n = 306)** (> +0.75 D)	2.67(2.04)	22.66(0.93)	43.88(1.40)	3.21(0.40)
p value for trend	< 0.001	< 0.001	0.31	<0.001

Results shown are the Mean (Standard Deviation; SD). D = Diopter. p values were obtained using one-way ANOVA. Data are for right eye, as there were no significant differences between the measurements of the right and left eyes.

### Genetic association for myopia and hypermetropia

**Figure 1:**
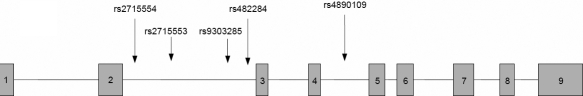
Schematic representation of the physical location of the 5 tag single nucleotide polymorphisms within the *RARA* gene. The grey boxes represent exons and the lines represent introns. The tSNPs are identified by their reference numbers in dbSNP.

A total of five tSNPs, which tagged 10 known SNPs with a MAF >5% in *RARA*, were genotyped in this study. All five tSNPs were in Hardy Weinberg Equilibrium, with the failure rate of genotyping being 2%. All five tSNPs were intronic, with 4 being located in intron 2, the longest intron of the gene (Figure 1). As expected, none of the tSNPs were in high LD with each other (r^2^ <0.8). Genotype frequencies were compared between the three cohorts but no significant differences were evident (Table 2). Genotype frequencies for these five SNPs were compared for the ‘myopia’ and ‘hypermetropia’ groups relative to the ‘emmetropia’ group. As can be seen in Table 3, no statistically significant associations were observed for these five tSNPs in the ‘myopia’ or the ‘hypermetropia’ cohort. In addition, we also undertook an analysis for each cohort separately, although it decreased the study power, the results were similar for each of the cohorts. An analysis was also performed for males and females separately and again no significant differences were observed.

**Table 2:** Demographic characterizes and genotype frequencies of 5 tag single nucleotide polymorphisms of participants from three different study population.

		**BMES, N=131**	**GEMs, N=570**	**VIP, N=101**	**p value**
Sex, Male, n(%)		40 (30.5)	212 (37.2)	39 (38.5)	0.32
Age, Mean (SD)		61.5 (8.6)	52.9 (13.6)*	61.6 (10.1)	<0.001
Genotypes					
rs2715554	TT	90 (69.2)	392 (69.1)	70 (71.6)	0.56
	TC	33 (25.4)	159 (28.0)	26 (26.3)	
	CC	7 (5.4)	16 (2.8)	2 (2.1)	0.10
rs2715553	TT	50 (38.2)	169 (29.7)	26 (26.6)	
	TC	56 (42.7)	286 (50.3)	59 (60.1)	
	CC	25 (19.1)	114 (20.0)	13 (13.3)	
rs9303285	TT	93 (72.1)	437 (77.1)	78 (80.9)	0.44
	TC	31 (24.0)	118 (20.8)	16 (16.4)	
	CC	5 (3.9)	12 (2.1)	3 (3.1)	
rs482284	GG	65 (50.0)	292 (51.8)	45 (46.8)	0.02**
	GA	44 (33.8)	229 (40.6)	45 (46.8)	
	AA	21 (16.2)	43 (7.6)	6 (6.4)	
rs4890109	GG	116 (89.2)	507 (88.8)	86 (87.5)	0.90
	GT	13 (10.0)	61 (10.7)	12 (12.5)	
	TT	1 (0.8)	3 (0.5)	0 (0)	

The asterisk denotes participants only from GEM study were younger than the other two studies. The double asterisk indicates that after Bonferoni correction, the corrected p value (0.05/5) = 0.01 to obtain a significant difference amongst the three studies for the five tag single nucleotide polymorphisms

**Table 3:** Association analysis of myopia or hypermetropia compared to emmetropia with the 5 *RARA* tag single nucleotide polymorphisms.

**tSNP**	**Genotype**	**Frequency emmetropia n (%)**	**Frequency myopia n(%)**	**p value**	**Frequency hypermetropia n (%)**	**p value**
rs2715554	TT	79 (69.3)	258 (69.0)	0.17	213 (70.1)	0.21
	TC	28 (24.6)	106 (28.3)		83 (27.3)	
	CC	7 (6.1)	10 (2.7)		8 (2.6)	
rs2715553	TT	33 (28.7)	114 (30.3)	0.46	97 (31.9)	0.42
	TC	64 (55.7)	187 (49.7)		148 (48.7)	
	CC	18 (15.7)	75 (19.9)		59 (19.4)	
rs9303285	TT	91 (79.8)	290 (77.7)	0.81	225 (74.3)	0.49
	TC	20 (17.5)	75 (20.1)		69 (22.8)	
	CC	3 (2.6)	8 (2.1)		9 (3.0)	
rs482284	GG	59 (52.7)	197 (52.8)	0.95	145 (47.9)	0.68
	GA	43 (38.4)	146 (39.1)		128 (42.2)	
	AA	10 (8.9)	30 (8.0)		30 (9.9)	
rs4890109	GG	104 (90.4)	335 (88.4)	0.58	268 (88.4)	0.73
	GT	11 (9.6)	41 (10.8)		34 (11.2)	
	TT	0 (0.0)	3 (0.8)		1 (0.3)	

The p value indicates the significant level from χ^2^ tests in comparison with emmetropia.

**Figure 2:**
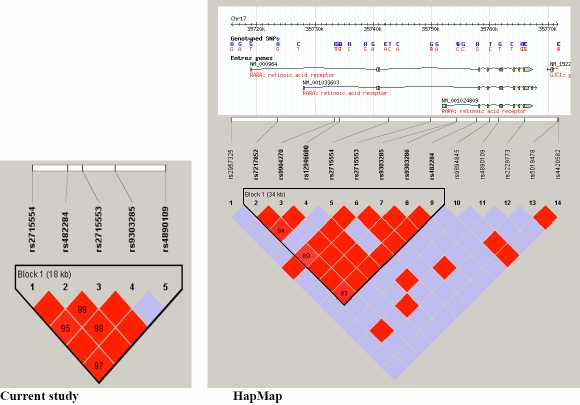
Linkage disequilibrium (LD) map comparing the current study with HapMap data for single nucleotide polymorphisms in the *RARA* gene. In the left hand panel is shown the LD block with r^2^ values indicated in the red diamonds and the position of the 5 tag SNPs for the current study. In the right hand panel the LD map from available HapMap data with position of available SNPs as well as the 5 tag SNPs (r^2^ values are indicated in the diamonds).  At the top of the panel are shown the different *RARA* alternatively spliced transcripts at this location on chromosome 17 (http://genome.ucsc.edu/).

All five SNPs for the population under study where in high LD with each other (r2 <0.95) with the exception of rs4890109 which is not in high lD with rs9303285. All five SNPs studies in this population fall within the same LD block. This is comparable, but not identical , to the HapMap data from the CEU population which places rs4890109 in a different LD block to the other four SNPs (Figure 2). Haplotype analysis was undertaken using a two, three, four, or five sliding SNP window to investigate haplotype associations. No significant associations were observed based on this analysis and was similar to that obtained from analysis when using single SNPs.

### Genetic association for refraction and ocular biometry

Although refractive measures were available for all 802 individuals, ocular biometry was only available for 593 subjects. Ocular biometry measures showed a normal distribution and were each assessed for genetic association using quantitative analysis where the mean values of the three genotypes for each of the five tSNPs were compared and a p-value calculated. None of the five tSNPs compared showed statistically significant associations for axial length, corneal curvature or anterior chamber depth as shown in Table 4. The tSNP rs482284 (5’ of exon 3) initially showed significant association with axial length (p=0.04) but this association did not remain significant after Bonferroni correction.

**Table 4:** Quantitative association analysis of ocular biometric measurements with the 5 *RARA* tag single nucleotide polymorphisms.

**Genotypes**	**Frequency**	**Axial length, mean (SD)**	**Anterior chamber depth, mean (SD)**	**Corneal curvature mean (SD)**
rs2715554				
TT	410	23.98 (1.72)	3.41 (0.43)	44.06 (1.35)
TC	176	24.06 (1.65)	3.38 (0.43)	43.94 (1.60)
CC	21	23.90 (2.59)	3.29 (0.41)	44.21 (1.73)
p value		0.84	0.23	0.66
rs2715553				
TT	188	23.92 (1.85)	3.36 (0.46)	44.01 (1.66)
TC	306	24.07 (1.69)	3.42 (0.42)	43.99 (1.35)
CC	115	23.97 (1.70)	3.42 (0.44)	44.18 (1.28)
p value		0.79	0.27	0.34
rs9303285				
TT	464	24.03 (1.74)	3.40 (0.44)	44.05 (1.39)
TC	129	24.01 (1.77)	3.41 (0.42)	43.99 (1.56)
CC	12	23.25 (1.50)	3.25 (0.29)	43.97 (1.86)
p value		0.13	0.23	0.86
rs482284				
GG	309	24.07 (1.78)	3.41 (0.43)	43.97 (1.40)
GA	245	24.03 (1.68)	3.42 (0.43)	44.14 (1.48)
AA	50	23.52 (1.76)	3.27 (0.44)	43.93 (1.50)
p value		0.04	0.05	0.87
rs4890109				
GG	539	24.01 (1.75)	3.40 (0.44)	44.05 (1.39)
GT	68	23.99 (1.70)	3.41 (0.44)	44.0 (1.77)
TT	4	24.57 (0.90)	3.31 (0.49)	43.93 (2.15)
p value		0.53	0.67	0.87

The p value indicates the significant level from the test of one-way ANOVA.

## Discussion

Our study represents the first to assess for a genetic association between *RARA* and myopia, hypermetropia, and ocular biometry. Using a tag SNP approach we were able to capture all common genetic variants within the coding and promoter regions of *RARA* by genotyping five tSNPs. We found no association with myopia and hypermetropia with these five tSNPs in our Caucasian cohort. We also performed a quantitative analysis using axial length, corneal curvature and anterior chamber depth and again found no association of these traits with the five tSNPs. These findings present strong evidence that any effect that *RARA* has shown in animal models is unlikely to be due to the presence of DNA variants within the gene or associated regions.

Although our findings do not implicate a direct genetic role for *RARA* in myopia and hypermetropia, we cannot rule out the possibility that *RARA* may be just one link in a yet unknown complex pathway involved in causing refractive errors. There is strong evidence from animal studies for a role of *RARA* and retinoic acid in the development of myopia as well as it having a role in regulating eye length (axial length) [42,46,53]. It has been shown that the introduction of retinoic acid to the diet of chicks in form-deprivation experiments resulted in an overall increase in eye length and conversely, inhibition of the ligand was shown to have the opposite effect [42,54]. In addition, mice lacking both copies of *RARA* presented with a lower eye weight and reduced retinal areas compared to wild-type mice [46]. However, our study suggests that any putative biological role that *RARA* might have on the development of refraction is unlikely to be mediated by common variations in the DNA sequence. We cannot rule out the possibility that rare genetic variants or variants resulting in small effect size might contribute to changes in refraction. Alternative mechanisms of action such as those mediated by epigenetic effects or those that affect gene expression may play a role and this needs to be further explored to fully understand what role, if any, *RARA* has in the development of refractive errors.

Our study cohort has been carefully selected to encompass a homogenous population with clear phenotypic definitions. We selected subjects with only Caucasian ethnicity to minimize population admixture and used strict definitions of refractive error to prevent misclassification. It has been suggested that extreme sampling provides a powerful method to improve the power of genetic association studies [55,56]. In the current study we recruited individuals across the entire spectrum of refractive error from hypermetropia to those with high myopia. This provided us with the advantage that both refractive (qualitative) and ocular biometry measures (quantitative) could be analyzed, giving a more thorough analysis of refraction and its underlying determinants. Utilizing a tSNP approach has also strengthened our study and has ensured maximal genetic coverage of the *RARA* gene. However, the tSNP approach also has limitations in that only common tSNPs (MAF <5%) were genotyped, so there remains a possibility that we have missed rarer alleles in *RARA* that might contribute to the development of refractive errors such as myopia and hypermetropia [57]. As with any case-control association study, particularly one with negative results, there are always questions of power to detect association and cohort size. The power calculations that we performed suggest that this cohort is sufficiently large to detect potential changes associated with *RARA* but we cannot rule out the possibility that smaller effect changes in *RARA* may have been missed by using a cohort of this size.

This study has found that *RARA* is not genetically associated with myopia or hypermetropia despite its biological role in the eye. Although this is a negative result, additional validation work is still required to assess for rare variants and to assess for association in larger cohorts. Additional research exploring the possible role for *RARA* in the development of refractive errors via mechanisms that do not involve direct changes in the nucleotide sequence is also warranted.
